# Efficiency and Mechanism of Sulfamethoxazole Removal via Peroxymonosulfate Activation by Using Base Etched Montmorillonite

**DOI:** 10.3390/molecules31152715

**Published:** 2026-08-04

**Authors:** Chen Tian, Yuchen Zhang, Chu Dai, Jing Li, Ting Liu, Jianwu Chen, Zhenye Liu, Jinhua Gan

**Affiliations:** 1 Yangtze River Fisheries Research Institute of Chinese Academy of Fishery Sciences, Wuhan 430223, China; 2 Freshwater Fisheries Research Center, Chinese Academy of Fishery Sciences, Wuxi 214081, China; 3 Center of Agro-Product Safety and Quality, Ministry of Agriculture and Rural Affairs, Beijing 100020, China; 4 Collage of Advanced Energy Materials, Hubei University of Education, Wuhan 430205, China; 5 Hubei Academy of Agricultural Sciences, Wuhan 430205, China

**Keywords:** peroxymonosulfate, base-etched montmorillonite, sulfamethoxazole, lewis basic sites, singlet oxygen

## Abstract

Developing efficient and eco-friendly catalysts for peroxymonosulfate (PMS) activation to degrade persistent antibiotics in water remains a major challenge. Herein, acid-base etched montmorillonite is employed to activate PMS for sulfamethoxazole (SMX) degradation under neutral to weakly alkaline conditions. Compared with pristine montmorillonite and acid-etched montmorillonite (A-Mon), base-etched montmorillonite (B-Mon) achieves the highest removal efficiency, eliminating 92% of SMX within 120 min. The degradation rate constant of the B-Mon/PMS system is 0.19 min^−1^, which is 2.7 times that of the pristine montmorillonite/PMS system. Reactive species analysis reveals that the B-Mon/PMS system increases the singlet oxygen (^1^O_2_) concentration by 10.3-fold compared to PMS alone. Electron spin resonance (ESR), temperature-programmed desorption (TPD), and quenching experiments demonstrate that medium-weak Lewis basic sites on the B-Mon surface play a pivotal role in PMS activation and SMX degradation. Moreover, these medium-weak Lewis basic sites facilitate ^1^O_2_ generation via the self-decomposition mechanism of PMS. Stability tests confirm that B-Mon exhibits excellent cycling stability and anti-interference capability. This work provides a deeper mechanistic insight into the development of environmentally friendly aluminum-based catalysts for practical remediation of complex water.

## 1. Introduction

In the past decades, extensive consumption of antibiotics in human clinical treatment and agricultural production has resulted in abundant residual antibiotics discharged into environmental compartments, raising increasing public concerns owing to their emerging adverse health outcomes including diseases and mortality [1,2]. Sulfamethoxazole, one of the most prevalently prescribed and consumed antibacterial agents, has been ubiquitously detected in diverse environmental matrices worldwide [3,4]. Existing investigations have verified that SMX exhibits low elimination efficiency during conventional water treatment and possesses high detection frequencies in aquatic ecosystems [5,6]. Restricted by the inferior removal performance of conventional wastewater treatment infrastructures and inherent recalcitrance against abiotic/biotic degradation in aqueous media, substantial quantities of SMX eventually infiltrate into aquatic environments [7,8]. Furthermore, residual SMX imposes detrimental impacts on human health, animal ontogeny and terrestrial/aquatic plant growth [9,10]. Accordingly, it is imperative to develop eco-friendly and cost-competitive remediation technologies to transform SMX and analogous hazardous contaminants into nontoxic derivatives prior to wastewater discharge into natural environments.

Peroxymonosulfate (PMS) has emerged as a promising candidate for environmental remediation, attributed to its capability of generating diverse reactive oxygen species (ROS, e.g., OH^•^, SO_4_^•−^ and ^1^O_2_), along with prominent operational superiority and moderate reagent cost [11,12]. It is widely acknowledged that transition metal-mediated PMS activation dominantly yields OH^•^ and SO_4_^•−^, and the cyclic redox conversion between high-valence and low-valence metal species is the core determinant governing radical production efficiency [13,14]. Lan and co-workers fabricated bimetallic CuCo_2_O_4_ catalysts; the incorporation of Cu cations remarkably accelerated SMX degradation and facilitated the reversible redox cycling between Co^2+^ and Co^3+^ [15]. Li and co-workers modulated cobalt stoichiometry in spinel Co_3-x_Mn_x_O_4_, which strengthened Mn(VI)-PMS interaction and accelerated Mn(III) regeneration. The optimized catalysts achieved efficient elimination and mineralization of organic pollutants with substantially improved catalytic durability [16]. Nevertheless, hetero-transition metal doping inevitably increases synthetic expenditure and triggers secondary pollution originating from toxic metal leaching, severely hindering the practical engineering application of such catalytic systems.

Aluminum-based mineral materials have attracted growing research interest in water pollution remediation, owing to their inherent advantages of low cost, high structural stability, and environmental compatibility. Dionysiou et al. [17] demonstrated that the Fe-O-Fe structure in Fe-Al layered double hydroxides (LDHs) significantly accelerates the Fe(III)/Fe(II) redox cycle, thereby greatly enhancing the degradation efficiency of bisphenol A. In a follow-up study, the same group incorporated main-group metals (e.g., Mg and Al) into the CuO lattice, which modulated the surface electron distribution of the catalyst and constructed dual reaction centers with electron-rich and electron-deficient characteristics. In this catalytic system, peroxymonosulfate (PMS) accepts electrons at electron-rich centers to generate OH^•^ and SO_4_^•−^, while bisphenol A adsorbs preferentially at electron-deficient centers, driving directional electron transfer from the pollutant to PMS [18]. Zhu et al. [19] first applied pristine MgAl-LDH as an ozonation catalyst and reported that it achieved 68% chemical oxygen demand (COD) removal for 2,4-dichlorophenoxyacetic acid (2,4-D), which was markedly superior to that of conventional FeOOH (50%). They further verified that surface Lewis acid-base sites on LDHs dominate the generation of non-radical reactive species during catalytic ozonation. Tian et al. [20] regulated the Mg/Al ratio of magnesium aluminate spinel and revealed that intrinsic Lewis basic sites promote the self-decomposition of PMS to yield ^1^O_2_, whereas oxygen-vacancy-induced Lewis basic sites activate PMS to generate a minor fraction of free radicals. Meanwhile, Lewis acid sites act as adsorption and enrichment centers for both PMS and sulfamethoxazole (SMX), serving as localized reaction platforms. Collectively, these findings demonstrate that aluminum-based catalysts exhibit great potential for the targeted regulation of reactive oxygen species (ROS), effectively circumventing the secondary pollution and high cost associated with metal leaching from conventional transition-metal catalysts.

Montmorillonite, a typical aluminum-bearing clay mineral with unsaturated coordination sites on its edge surfaces, naturally possesses intrinsic Lewis acid sites. Several studies have utilized these surface Lewis acid sites to boost catalytic performance in advanced oxidation processes [21,22]. Building on these prior findings, this work aims to elucidate the correlation between surface Lewis acid-base sites of montmorillonite and its PMS activation performance for antibiotic pollutant removal. Acid-base etching is a facile, reliable strategy for catalyst modification: it induces structural disruption and reconstruction without introducing exogenous metal elements, thereby enhancing catalytic activity and expanding the application scope of clay-based minerals in environmental remediation [23,24].

Accordingly, in this study, natural montmorillonite was used as a precursor and modified via controlled-temperature acid and alkali etching to obtain a series of modified montmorillonite catalysts, with the goal of enhancing the catalytic degradation efficiency of SMX. The microstructures, crystalline phases, and surface functional groups of the as-prepared catalysts were systematically characterized via scanning electron microscopy (SEM), X-ray diffraction (XRD), and Fourier-transform infrared spectroscopy (FTIR). The structural stability and reusability of the catalysts were assessed via FTIR spectroscopy, thermogravimetric (TG) analysis, SEM observation, and consecutive cycling experiments. Furthermore, a combination of quenching experiments, electron paramagnetic resonance (EPR) spectroscopy, CO_2_/NH_3_ temperature-programmed desorption (TPD), and quantitative ROS probing was employed to clarify the mechanism underlying PMS activation by alkali-treated montmorillonite surface sites and the associated ROS generation pathways. Finally, the degradation intermediates of SMX were identified, and a plausible degradation transformation pathway was proposed. The findings of this work provide new insights into the directional regulation of oxidative species generation via alkali-treated montmorillonite-activated PMS systems for efficient antibiotic removal from aqueous environments.

## 2. Results and Discussion

### 2.1. Structure Characterization of Different Montmorillonite Catalysts

**Figure 1:**
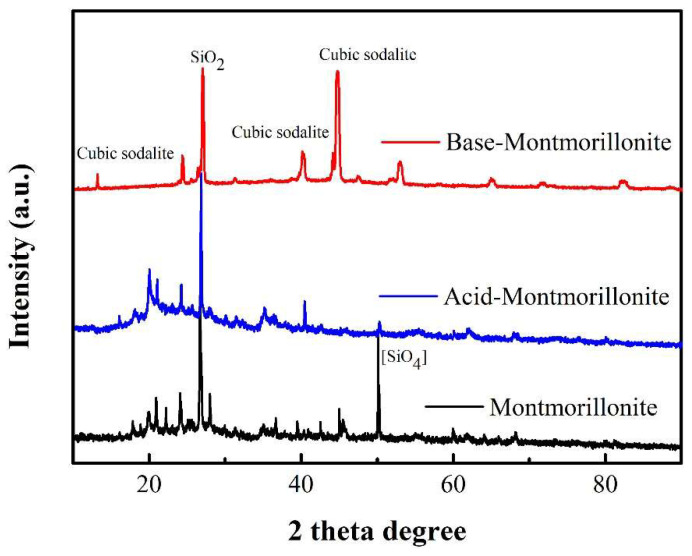
XRD patterns of montmorillonite, A-Mon and B-Mon, respectively.

The phase structures of the three montmorillonite samples were characterized by X-ray diffraction (XRD). As shown in Figure 1, XRD patterns were collected over a 2θ range of 10–90°. The pristine montmorillonite had a purity of approximately 92%, consistent with quantitative XRD analysis, and exhibited minor diffraction peaks corresponding to quartz impurities [25]. Specifically, the diffraction peak located at 22–25° is the characteristic main peak of montmorillonite [26]. Its disappearance after alkali treatment suggests the disruption of the ordered layered aluminosilicate framework. The leached Al and Si species are reconstructed into amorphous aluminosilicate, which offers abundant Lewis basic sites. The peak near 50° serves as an indicator for the intact siloxane tetrahedral framework and vanishes after both acid and alkali treatments. Acid treatment selectively destroys aluminum octahedra and induces the collapse of the 2:1 layered structure. By contrast, alkaline etching breaks down the tetrahedral lattice and eliminates the periodic structure corresponding to high-angle crystal planes. Furthermore, the newly emerging sharp peaks at approximately 14.5°, 40° and 45° after alkali treatment are assigned to cubic sodalite, which originates from the recrystallization of leached Si/Al species in the presence of Na^+^ [27].

**Figure 2:**
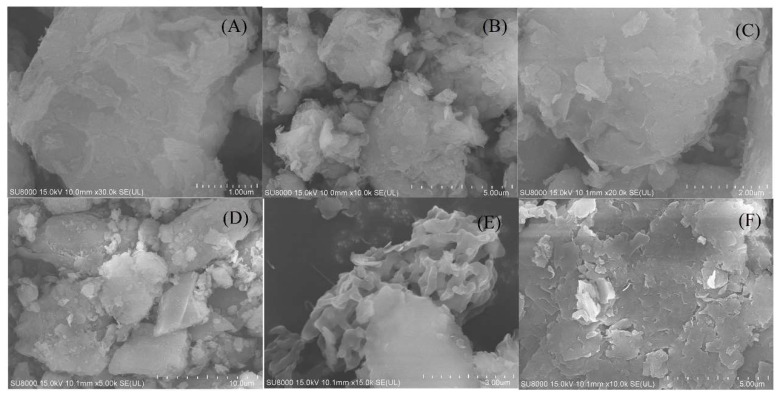
SEM images of (**A**,**B**) montmorillonite, (**C**,**D**) A-Mon and (**E**,**F**) B-Mon, respectively.

The morphologies of the three montmorillonite samples were examined by scanning electron microscopy (SEM). As shown in Figure 2A,B, the pristine montmorillonite exhibited a well-stacked lamellar structure composed of thin flakes, with no visible pores or cavities, and its layered architecture remained largely intact. Further analysis revealed that the pristine montmorillonite possessed a multilayered structure with flake diameters ranging from approximately 3 to 5 μm. As shown in Figure 2C,D, the acid-treated montmorillonite did not undergo pronounced morphological changes, although some flakes became disordered in stacking. As illustrated in Figure 2E,F, the alkali-treated montmorillonite displayed disordered stacking of flakes together with abundant heterogeneous pores. This observation indicates that Al and/or Si in the montmorillonite framework were dissolved and etched by OH^−^ ions from the alkaline solution. Therefore, it can be preliminarily concluded that the original layered structure of montmorillonite was disrupted after alkali activation. The specific chemical species leached or structural moieties damaged during this process remain to be further elucidated.

The chemical compositions of the three montmorillonite samples were analyzed by inductively coupled plasma optical emission spectrometry (ICP-OES), and their surface acid-base properties were characterized by temperature-programmed desorption (TPD). The detailed results are presented in Table 1. The pristine montmorillonite contained 5.6 wt% Al and 34.5 wt% Si. After acid treatment, the Al/Si ratio showed no significant change. In contrast, alkali treatment resulted in an approximately 3 percentage point increase in Al content and a corresponding 6 percentage point decrease in Si content. These findings indicate that Si was preferentially leached from the montmorillonite framework upon alkali treatment, exposing more AlO_6_ units on the edge surfaces and thereby altering the abundance of Lewis acid and base sites. TPD measurements were performed to quantify the changes in surface acid-base sites.

**Figure 3:**
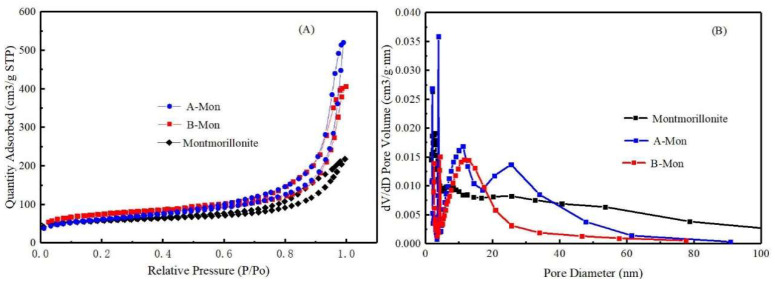
(**A**) Nitrogen adsorption–desorption isotherms and (**B**) pore size distribution curves of the three montmorillonite samples.

As presented in Figure 3 and Table S1, montmorillonite has a specific surface area of 221.32 m^2^/g. The specific surface area of A-Mon is comparable to that of montmorillonite, while B-Mon shows an increase of 67.3 m^2^/g. Both pore size and pore volume are increased after acid and alkali modification, with A-Mon achieving the highest values. Combined with the degradation results, it is concluded that specific surface area contributes moderately to the catalytic activity, whereas the variation in surface Lewis acid-base sites is the major influencing factor.

**Figure 4:**
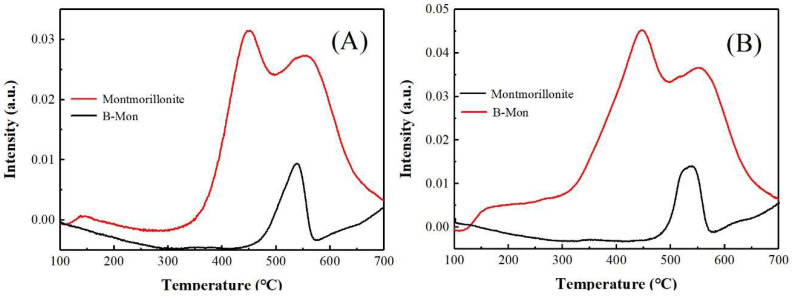
The results of NH_3_-TPD (**A**) and CO_2_-TPD (**B**) over different catalysts.

As shown in Figure 4A, the pristine montmorillonite exhibited a characteristic desorption peak at 500 °C, corresponding to strong Lewis acid sites with an acidity of 76.81 mmol/g. After alkali activation, the intensity of this peak decreased significantly, and the Lewis acidity dropped to 18.22 mmol/g. Similarly, as shown in Figure 4B, the pristine montmorillonite displayed a desorption peak at 520 °C, corresponding to Lewis basic sites with a basicity of 20.42 mmol/g [28,29]. The alkali-treated montmorillonite exhibited two characteristic desorption peaks with markedly enhanced intensity, and its total Lewis basicity increased to 90.24 mmol/g. Collectively, these results confirm that alkali activation substantially increased the abundance of strong basic sites while drastically reducing the density of Lewis acid sites. This is consistent with the fact that Si atoms in the Si-O bonds of montmorillonite act as Lewis acid sites; alkali-induced leaching of Si consequently diminished the Lewis acidity of the mineral.

### 2.2. Performance of Alkali-Treated Montmorillonite-Activated PMS

To evaluate the catalytic performance of the three montmorillonite samples, sulfamethoxazole (SMX) was selected as the model pollutant to assess their peroxymonosulfate (PMS) activation capability. It has been previously reported that PMS can generate non-radical reactive species under weakly alkaline conditions, which contribute to pollutant degradation.

**Figure 5:**
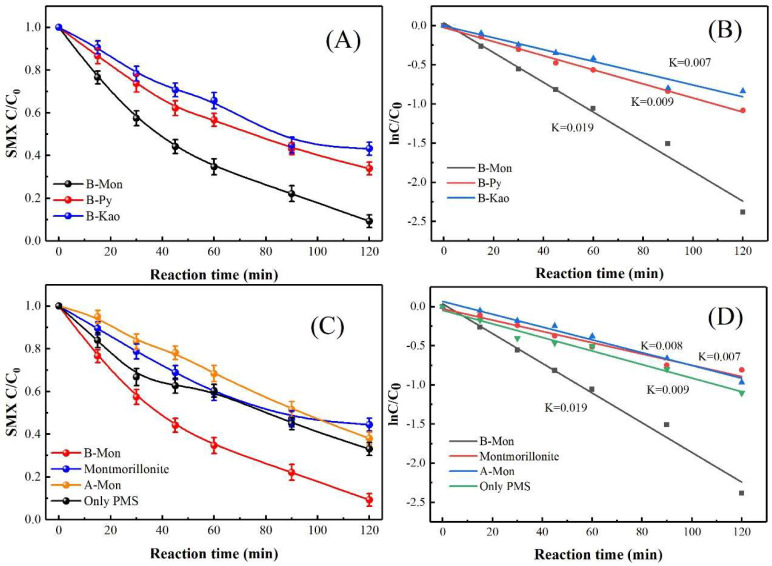
(**A**) Degradation efficiency of SMX by alkali-activated clay aluminum; (**B**) corresponding reaction rate curves; (**C**) degradation efficiency of SMX by modified montmorillonite; (**D**) corresponding reaction rate curves. K represents the pseudo-first-order kinetic rate constant.

The SMX degradation efficiencies of three alkali-activated clay minerals (montmorillonite, pyrophyllite, and kaolin) were first compared (Figure 5A). The results showed that the degradation efficiencies achieved with alkali-activated pyrophyllite and kaolin were comparable to that obtained with PMS alone. Further comparison revealed that the degradation efficiencies of the three alkali-activated clay minerals followed the order: montmorillonite > pyrophyllite > kaolinte. As shown in Figure 5C, treatment with PMS alone achieved approximately 67% SMX degradation in aqueous solution within 120 min. Among the three montmorillonite samples, pristine and acid-treated montmorillonite exhibited no significant difference in SMX degradation efficiency. In contrast, when alkali-treated montmorillonite was combined with PMS under weakly alkaline conditions, the SMX removal efficiency exceeded 92%. As shown in Figure S1, the B-Mon catalytic system achieves a TOC removal efficiency of approximately 60% for SMX within 120 min, indicating that the catalyst possesses favorable mineralization capacity. As listed in Table S2, a comparison of clay mineral catalysts and Co-based catalysts for SMX degradation was conducted in terms of degradation efficiency, catalyst dosage, PMS consumption, reaction parameters and reusability [30,31,32,33]. B-Mon enables fast SMX elimination. It requires less catalyst and PMS than Co-based catalysts. Although its degradation performance is similar to other clay materials, B-Mon is free from heavy metal leaching and subsequent secondary pollution. Meanwhile, the facile availability of B-Mon significantly cuts synthesis costs. Accordingly, B-Mon is a promising candidate for practical environmental applications.

**Figure 6:**
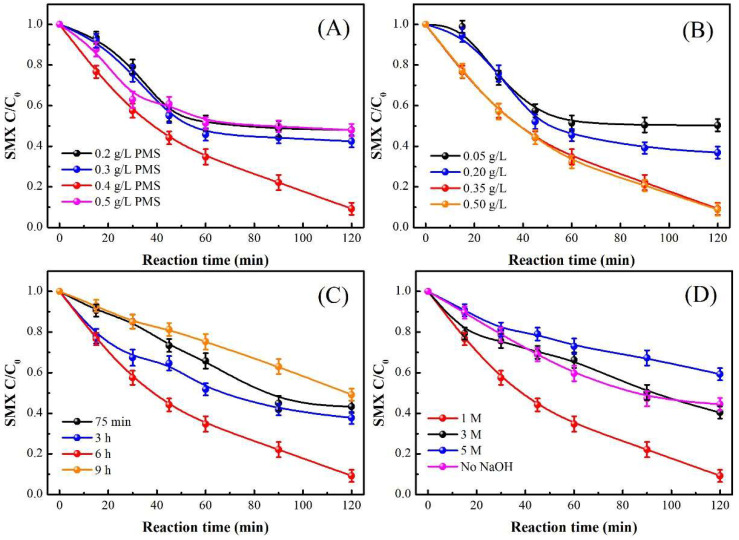
(**A**) PMS dosage in the alkali-treated montmorillonite/PMS system; (**B**) optimization of catalyst (alkali-treated montmorillonite) dosage; (**C**) effect of alkali-treated montmorillonite reaction time on SMX degradation efficiency; (**D**) effect of alkali concentration on SMX degradation efficiency.

The operational parameters of the alkali-treated montmorillonite/PMS system were subsequently optimized, starting with the dosages of catalyst and PMS. As shown in Figure 6A and Figure S2A, SMX degradation efficiency was dependent on PMS dosage at a given catalyst dosage. A maximum degradation efficiency of 92% within 120 min was achieved at a PMS dosage of 0.4 g/L, whereas further increases in PMS dosage resulted in diminished degradation efficiency. The decline in SMX degradation efficiency at PMS dosages above 0.4 g/L is mainly attributed to two mechanisms. Firstly, excess PMS quenches reactive oxygen species (ROS). Surplus HSO_5_^−^ reacts with the generated ROS: it consumes the dominant ^1^O_2_ via side reactions, and transforms trace SO_4_•^−^ and •OH into weakly oxidizing SO_5_•^−^, reducing the effective ROS concentration available for SMX degradation [34,35]. Secondly, overdosed PMS competitively occupies catalytic active sites. The alkali-etched montmorillonite activates PMS primarily through surface Lewis basic sites. Excess HSO_5_^−^ adsorbs onto these limited sites, lowering the contact probability of SMX with active centers and causing premature consumption of locally generated ROS [36,37]. This site saturation effect further restricts degradation performance. Therefore, the optimal PMS dosage was determined to be 0.4 g/L based on the maximum catalytic performance.

Figure 6B and Figure S2B shows SMX degradation efficiency as a function of catalyst dosage at the optimal PMS dosage. A SMX removal efficiency of 92% was achieved at a catalyst dosage of 0.35 g/L. When the catalyst dosage exceeds 0.35 g/L, SMX degradation efficiency declines instead of further increasing, which is mainly attributed to excess montmorillonite particles tend to aggregate at high loading, reducing the number of effectively exposed Lewis basic active sites and increasing mass transfer resistance in the suspension, thus limiting the contact of SMX and PMS with catalytic active centers [38].

Subsequently, the effects of alkali activation time and NaOH concentration on SMX degradation were investigated. As illustrated in Figure 6C, the optimal activation time was 6 h; prolonging the activation time beyond 6 h caused excessive structural damage to the montmorillonite framework, thereby impairing its catalytic activity. Further investigation into NaOH concentration (Figure 6D) revealed that 92% SMX removal was achieved within 120 min at a NaOH concentration of 1 M. An excessively low NaOH concentration failed to induce sufficient structural modification of montmorillonite, whereas an excessively high concentration altered the Al/Si ratio and triggered structural collapse. Accordingly, montmorillonite activated with 1 M NaOH for 6 h was employed for subsequent PMS activation and SMX degradation experiments.

### 2.3. Mechanistic Insight into the Degradation of SMX by B-Mon/PMS System

#### 2.3.1. Identification and Contribution Analysis of Reactive Oxygen Species in PMS Activation by Alkali-Treated Montmorillonite

**Figure 7:**
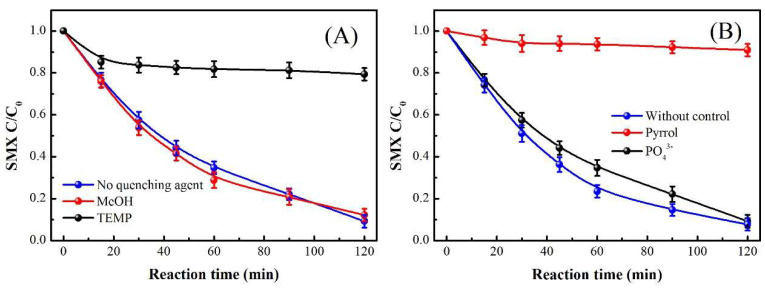
Effect of (**A**) two typical quenchers and (**B**) two Lewis acid–base site inhibitors on the degradation efficiency of SMX by the alkali-treated montmorillonite/PMS system.

The above results demonstrate that alkali-treated montmorillonite exhibits superior catalytic performance in SMX degradation, and the Lewis base sites exposed on its edge surfaces are critical for this enhanced activity. It is therefore essential to elucidate the correlation between surface active sites and the reactive oxygen species (ROS) generated in the catalytic system. Methanol (MeOH, a common quencher for both OH^•^ and SO_4_^•−^) and 2,2,6,6-tetramethyl-4-piperidinol (TEMP, a selective trapping agent for ^1^O_2_) were employed to identify the dominant ROS [39,40,41]. As shown in Figure 7A, the addition of MeOH to the alkali-treated montmorillonite/PMS system exerted a negligible inhibitory effect on SMX degradation, whereas TEMP reduced the degradation efficiency by approximately 80%, indicating that ^1^O_2_ is the primary reactive species responsible for SMX removal.

To further verify the role of surface-active sites, phosphate (a Lewis acid site blocker) and pyrrole (a selective Lewis base site quencher) were introduced into the reaction system [42,43]. As shown in Figure 7B, the presence of 1 mM phosphate did not significantly alter SMX removal efficiency; however, the addition of 1 mM pyrrole decreased the SMX removal efficiency drastically from 92% to 3%. These findings confirm that the Lewis base sites exposed on the mineral edge surfaces play a dominant role in the catalytic process, and that singlet oxygen (^1^O_2_) is the predominant ROS, contributing 80% to the overall SMX degradation efficiency, which is consistent with the TEMP quenching results.

**Figure 8:**
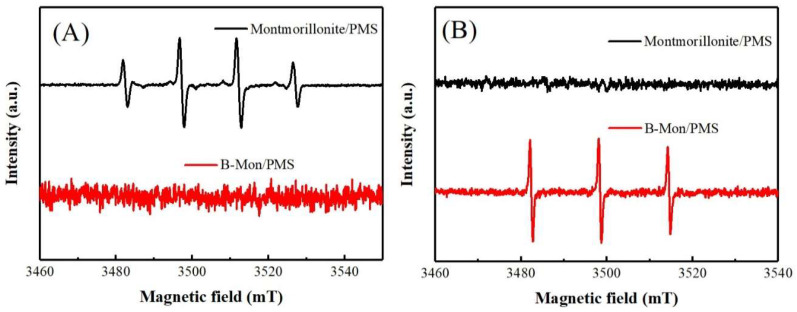
ESR spectra of the alkali-treated montmorillonite/PMS system recorded during the reaction using DMPO (**A**) and TEMP (**B**).

Electron paramagnetic resonance (EPR) spectroscopy was employed to directly confirm the ROS generated in both the pristine and B-Mon/PMS systems. As illustrated in Figure 8A, using 5,5-dimethyl-1-pyrroline N-oxide (DMPO) as a spin trap for free radicals, characteristic quartet signals of the DMPO-OH^•^ adduct were detected in the B-Mon/PMS system. In contrast, no discernible radical signals were observed in the alkali-treated montmorillonite/PMS system, confirming the absence of free radical species [44,45]. As shown in Figure 8B, TEMP-trapping experiments revealed a characteristic 1:1:1 triplet signal of the TEMP-^1^O_2_ adduct in the B-Mon/PMS system, with significantly higher intensity than that in the pristine system [46,47]. Collectively, these results demonstrate that alkali treatment of montmorillonite increases the abundance of Lewis base sites while reducing Lewis acid sites, shifting the PMS activation mechanism from a radical-dominated pathway to a purely non-radical pathway.

#### 2.3.2. Proposed PMS Activation Mechanism

**Figure 9:**
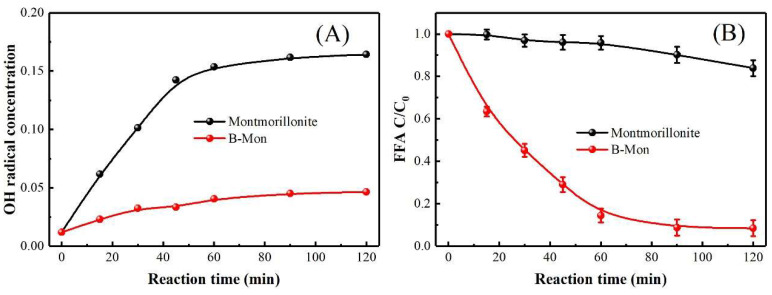
(**A**) Hydroxyl radical concentration profiles in different systems; (**B**) Singlet oxygen concentration profiles in different systems.

To quantitatively compare the generation of OH^•^ and ^1^O_2_ in the two systems, benzoic acid (BA) was first used as a molecular probe to quantify OH^•^ generation, based on the formation of hydroxylated products (primarily p-hydroxybenzoic acid). As shown in Figure 9A, the OH^•^ generation rate in the pristine montmorillonite/PMS system was 0.16 µmol/s, whereas it was only 0.05 µmol/s in the alkali-treated montmorillonite/PMS system, indicating that alkali treatment significantly suppressed hydroxyl radical generation.

Subsequently, furfuryl alcohol (FFA), a highly selective probe for ^1^O_2_, was employed to quantify singlet oxygen generation. As shown in Figure 9B, the pristine montmorillonite/PMS system degraded only 20% of FFA within 120 min, and the ^1^O_2_ generation yield was calculated as 0.31 × 10^−5^ mM^−1^ using the established pseudo-first-order kinetic model for ^1^O_2_-mediated FFA degradation. In contrast, the alkali-treated montmorillonite/PMS system achieved approximately 90% FFA removal, with a ^1^O_2_ generation yield of 3.2 × 10^−5^ mM^−1^. Notably, the ^1^O_2_ generation yield in the alkali-treated montmorillonite/PMS system was more than 10-fold higher than that in the pristine system, whereas OH^•^ generation was reduced by approximately 69% (a factor of ~3). This dramatic enhancement in ^1^O_2_ production is attributed to the abundant Lewis base sites exposed on alkali-treated montmorillonite, which is the key factor driving the accelerated SMX degradation.

**Figure 10:**
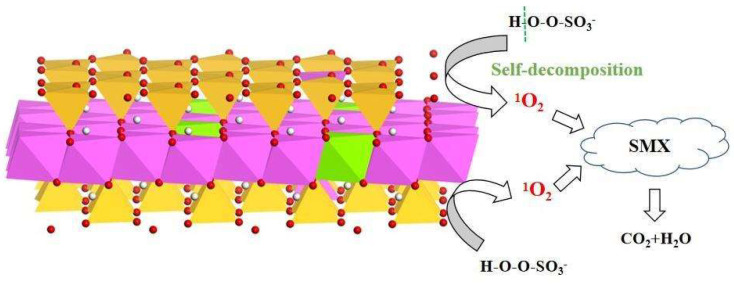
The proposed mechanism for the alkali-treated montmorillonite/PMS system.

Based on the above characterization and experimental results, a plausible PMS activation mechanism over B-Mon is proposed (Figure 10). In the B-Mon/PMS system, electrons are transferred from SMX (via its electron-donating groups) to the abundant Si-associated Lewis acid sites on the montmorillonite edge surfaces, which then activate PMS to generate a small number of free radicals (primarily OH^•^ and SO_4_^•−^). Owing to the low density of Lewis base sites on pristine montmorillonite, negligible ^1^O_2_ is produced via the non-radical pathway.

In contrast, alkali treatment preferentially leaches Si atoms from the montmorillonite framework, exposing abundant AlO_6_-derived Lewis base sites on the edge surfaces while simultaneously reducing the density of Lewis acid sites. In the alkali-treated montmorillonite/PMS system, these enriched Lewis base sites act as catalytic centers to promote the deprotonation and self-decomposition of PMS, which is the dominant pathway for ^1^O_2_ generation. Thus, ^1^O_2_-mediated non-radical oxidation becomes the exclusive pathway for SMX degradation. SMX molecules are then efficiently oxidized by ^1^O_2_, ultimately being mineralized into harmless inorganic species (e.g., CO_2_, H_2_O, SO_4_^2−^) or transformed into low-toxicity organic intermediates. The key reactions involved in this process are summarized in Equations (1)–(4).

Al-O + OH^−^ → Al-O^−^ + H_2_O (1)

HSO_5_^−^ + Al-O^−^ → SO_5_^2−^ + Al-OH (2)

HSO_5_^−^ + SO_5_^2−^ → HSO_4_^−^ + SO_4_^2−^ + ^1^O_2_ (3)

^1^O_2_ + SMX → O_2_ + Degradation products (4)

### 2.4. Stability of the B-Mon/PMS System

**Figure 11:**
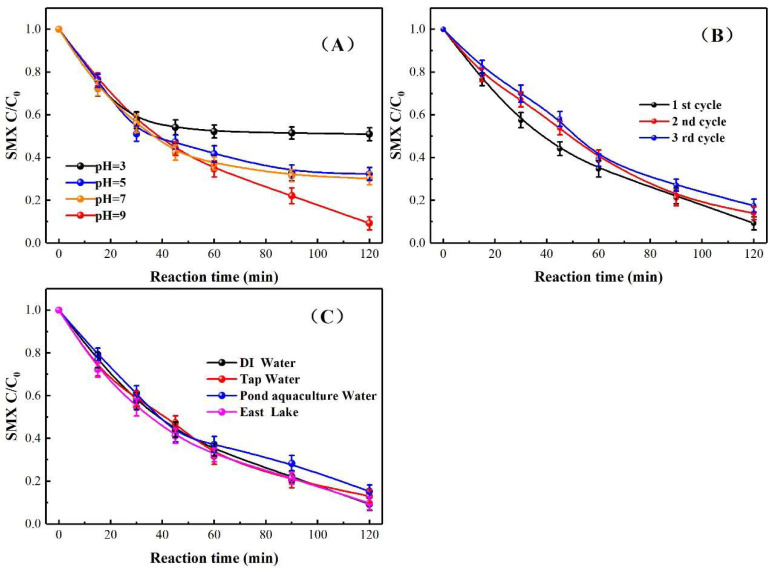
(**A**) SMX degradation efficiency of the alkali-treated montmorillonite/PMS system under different pH conditions; (**B**) cycling performance of the alkali-treated montmorillonite/PMS system; (**C**) SMX degradation efficiency of the alkali-treated montmorillonite/PMS system in different water matrices.

Subsequently, the influence of pH and the cyclic stability of the system were investigated. As shown in Figure 11A, the degradation efficiency of SMX was significantly affected by pH, indicating that the alkali-treated montmorillonite/PMS system achieves high SMX removal only under alkaline conditions. After evaluating the catalytic activity, the reusability of the catalyst was examined. As presented in Figure 11B, multiple-cycle experiments were conducted under identical experimental conditions. The results showed that although the SMX removal efficiency after three cycles slightly decreased compared with the first cycle (92.5% vs. 83.5%), the catalyst still exhibited favorable stability and high removal efficiency.

The degradation efficiency of the mineral catalyst in real water matrices is a crucial metric for evaluating its practical applicability. As shown in Figure 11C, four types of water sources deionized water, tap water, pond aquaculture water, and East Lake water were used to assess the SMX removal efficiency of the alkali-treated montmorillonite/PMS system. The results demonstrated that the SMX removal efficiency exceeded 90% in all tested water matrices. Overall, the alkali-treated montmorillonite exhibited excellent degradation efficiency in complex real water environments, good cyclic stability, and high catalytic degradation efficiency under weakly alkaline conditions. These findings substantially broaden the application of aluminosilicate minerals in PMS-based oxidation systems and provide a theoretical basis for future system design.

As shown in Figure S3 and Table S3, the contents of Al, Si and Mg in the catalyst gradually decrease with each cycle. Specifically, the Al concentration drops from 8.62 to 7.06 after three cycles. The continuous leaching of elemental components reduces the number of Lewis acid-base active sites on the catalyst surface. The insufficient Al-O bonds available for interaction with PMS and SMX are the main reason for the declined SMX degradation activity during cycling.

## 3. Experimental Methods

### 3.1. Reagents and Chemicals

Montmorillonite, HNO_3_, NaCl, Na(PO_4_)_3_, NaHCO_3_, NaOH, ethanol, urea and all pollutants reagents were purchased from Sinopharm Chemical Reagent Co., Ltd. Peroxymonosulfate (PMS, KHSO_5_⋅0.5KHSO_4_⋅0.5K_2_SO_4_), Na_2_S_2_O_3_ and 2,2,6,6-Tetramethyl-4-piperidinol (TEMP, 99%) were purchased from Sigma Aldrich (St. Louis, MO, USA). All reagents were used directly and without any purification.

### 3.2. Synthesis of Different Montmorillonite Catalysts

In a 100 mL round-bottom flask, 1 g of montmorillonite mineral was added and mixed with 60 mL of deionized water under magnetic stirring to form a homogeneously dispersed suspension. The pH was then adjusted using NaOH and HNO_3_ solutions of appropriate concentrations. After stirring for another 30 min, the suspension was reacted in an oil bath at 75 °C for 6 h. Upon completion of the reaction, the resulting product was collected, washed successively with deionized water and absolute ethanol until neutral, and then dried overnight in an oven at 60 °C. The final product was denoted as the modified montmorillonite material.

### 3.3. Characterization of Montmorillonite Catalysts

Powder X-ray diffraction (XRD) patterns were recorded by a Rigaku Multiflex diffractometer (Tokyo, Japan) with Cu Kα radiation over a 2θ range from 10 to 90°. Scanning electron microscope (SEM) images were studied using Hitachi SU8100 (Tokyo, Japan). The X-ray photoelectron spectroscopy (XPS) was performed with a MULTILAB2000 electron spectrometer from VG Scientific (West Sussex, UK) by using 300 W Al K*α* radiation (225 W, 15 mA, 15 kV). The surface acid or base property of different nFe-Py catalysts were measured by temperature programmed desorption (TPD). Inductively coupled plasma-mass spectrometry (ICP-OES, Thermo Fisher, Waltham, MA, USA) was applied for determination of iron metals in this work.

### 3.4. Degradation Experiments by PMS Activation over Montmorillonite Catalysts

All the experiments were conducted in a 100 mL beaker at room temperature. The catalytic activity was evaluated by the degradation of sulfamethoxazole (SMX). Unless otherwise specified, 30 mg montmorillonite catalysts were dispersed into 100 mL 10 mg/L SMX solution and then stirred about an hour to achieve adsorption equilibrium. Then the desired amount of PMS was added into the above suspension to start the degradation reaction. At a given time interval, 2 mL of water sample was withdrawn and quenched with 150 µL of Na_2_S_2_O_3_ solution and filtered by a 0.45 µm membrane filter for further analysis. The concentration of SMX was determined by a high-performance liquid chromatograph.

### 3.5. Reactive Oxygen Species Generation Experiments by PMS Activation over Montmorillonite Catalysts

Hydroxyl radical concentration was indirectly determined by measuring the concentration of p-benzoquinone in the system using the benzoic acid method. First, a standard curve of p-benzoquinone was constructed based on its corresponding concentrations. Then, the hydroxyl radical concentration was calculated using the appropriate formula. The specific experimental conditions were as follows: high-performance liquid chromatography (HPLC-Ultimate 3000, Thermo Fisher, Waltham, MA, USA) equipped with a UV detector and a C18 column [48,49]. The concentration of hydroxyl radicals generated during the catalytic process was calculated using Equation (5):

(5) Cummulative OH• produced = p − HBA × 5.87

The concentration of p-benzoquinone in the system was also determined using the p-hydroxybenzoic acid method to directly measure the sulfate radical concentration. First, a standard curve of p-benzoquinone was established based on its corresponding concentrations, and then the hydroxyl radical concentration was calculated using the relevant formula [50,51,52].

The concentration of singlet oxygen (^1^O_2_) in the system was determined using the furfuryl alcohol (FFA) method [53,54]. The specific experimental conditions were as follows: HPLC (Ultimate 3000, UV detector, C18 column). The mobile phase consisted of phosphoric acid aqueous solution (pH = 2.6) and acetonitrile in a 60:40 volume ratio, with a flow rate of 1.0 μL/min. The column oven temperature was 35 °C, the detection wavelength was 220 nm, and the injection volume was 10 μL. The steady-state singlet oxygen concentration and the formation potential during the reaction were calculated using Equations (6) and (7):

(6) O21 = lnFFA0FFAtk1O2,FFA

(7)  R1O2,form=O21sskd+k1O2,FFAFFA

where [^1^O_2_]_ss_ is the steady-state concentration of singlet oxygen, *k*_1O2,FFA_ is the reaction kinetic constant (1.2 × 10^8^ M^−1^ s^−1^), k_d_ = 2.7 × 10^4^ s^−1^, and R_1O2,form_ is the singlet oxygen formation potential [55,56].

## 4. Conclusions

Montmorillonite was modified via acid and alkali etching, and the performance of the resulting materials in activating PMS for pollutant degradation was investigated. It was found that the alkali-treated montmorillonite achieved over 92% SMX removal within 120 min. The alkali-treated montmorillonite/PMS system was minimally affected by environmental factors and exhibited excellent cyclic stability. XRD, SEM, ICP-OES, and TPD characterizations confirmed that the crystal structure of montmorillonite was altered upon alkali treatment: the framework Si serving as Lewis acid sites was leached out, exposing abundant -O^−^ groups that function as Lewis base sites. These exposed Lewis base sites are the key to enhancing SMX degradation efficiency. Quenching experiments and EPR results demonstrated that the dominant pathway for PMS activation by alkali-treated montmorillonite is a non-radical pathway. The amount of ^1^O_2_ generated by alkali-treated montmorillonite-activated PMS was over 10 times higher than that generated by pristine montmorillonite. The Lewis base sites on the mineral edge surfaces are the critical sites responsible for activating PMS to produce ^1^O_2_.

## Figures and Tables

**Table 1:** The chemical compositions of the three montmorillonite samples.

Samples	Acid Sites ^a^ (mmol/g)	Basic Sites ^a^ (mmol/g)	Al ^b^ (wt.%)	Si ^b^ (wt.%)	Mg ^b^ (wt.%)
montmorillonite	76.81	20.42	5.61	34.50	0.63
A-Mon	72.35	21.36	5.66	35.64	0.61
B-Mon	18.22	90.24	8.62	28.14	0.91

^a^ TPD; ^b^ ICP-OES.

## Data Availability

The data supporting the findings of this study are available from the corresponding author upon reasonable request. All data have been processed and analyzed as presented in the manuscript.

## Supplementary Materials

The following supporting information can be downloaded at: https://www.mdpi.com/article/10.3390/molecules31152715/s1, Figure S1. TOC in the B-Mon/PMS system; Figure S2. Corresponding reaction rate curves of (A) PMS dosage and (B) catalyst dosage in the B-Mon/PMS system; Figure S3. XRD patterns of before and after degradation; Table S1. Specific surface area and pore properties data of three montmorillonite samples; Table S2. Comparison of different conditions of B-Mon with other PMS activation catalysts; Table S3. The chemical compositions of the B-Mon/PMS system after degradation.
