# Supplementary material for: Efficiency and Mechanism of Sulfamethoxazole Removal via Peroxymonosulfate Activation by Using Base Etched Montmorillonite
Source: Molecules. 2026 Aug 4;31(15):2715. doi: 10.3390/molecules31152715 (PMC13468016; doi:10.3390/molecules31152715)
Supplement: Supplementary file 1 [file molecules-31-02715-s001.zip › molecules-4450663-supplementary.pdf]

Supplementary Information

Efficiency and Mechanism of Sulfamethoxazole Removal via Peroxymonosulfate Activation by Using Base Etched Montmorillonite

Chen Tian [1,2,*], Yuchen Zhang [3], Chu Dai [4], Jing Li [5], Ting Liu [1,2], Jianwu Chen [1,2], Zhenye Liu [1,2] and Jinhua Gan [1,2,*]

[1] Yangtze River Fisheries Research Institute of Chinese Academy of Fishery Sciences, Wuhan 430223, China
[2] Freshwater Fisheries Research Center, Chinese Academy of Fishery Sciences, Wuxi 214081, China
[3] Center of Agro-Product Safety and Quality, Ministry of Agriculture and Rural Affairs, Beijing 100020, China
[4] Collage of Advanced Energy Materials, Hubei University of Education, Wuhan 430205, China
[5] Hubei Academy of Agricultural Sciences, Wuhan 430205, China
* Correspondence: tianchen@yfi.ac.cn (C.T.); gjh@yfi.ac.cn (J.G.)

Contents

Table S1. Specific surface area and pore properties data of three montmorillonite samples.
Table S2. Comparison of different conditions of B-Mon with other PMS activation catalysts.
Table S3. The chemical compositions of the B-Mon/PMS system after degradation.
Figure S1. TOC in the B-Mon/PMS system.
Figure S2. Corresponding reaction rate curves of (A) PMS dosage and (B) catalyst dosage in the B-Mon/PMS system.
Figure S3. XRD patterns of before and after degradation.

Table S1. Specific surface area and pore properties data of three montmorillonite samples.

Samples	Specific Surface Area (m^2/g)	Pore Diameter (nm)	Pore Volume (cm^3/g)
Montmorillonite	213.82	6.31	0.34
A-Mon	222.47	14.50	0.81
B-Mon	281.12	9.00	0.63

Table S2. Comparison of different conditions of B-Mon with other PMS activation catalysts.

**Catalysts**	**Degradation rate (%)**	**Catalyst dosage(g/L)**	**PMS(g/L)**	**Reaction condition(min)**	**stability**	**Reference**
$CoMn_2O_4$	98	0.4	0.1 mM	40	3	[1]
MnCoO@HC	100	0.4	0.3	60	3	[2]
Fe-PMS-MC	100	1	-	360	-	[3]
$FeOOH/Al_2O_3$	93	0.15	0.2	120	4	[4]
B-Mon	92	0.35	0.4	120	3	This work

Table S3. The chemical compositions of the B-Mon/PMS system after degradation.

Cycles	Al (wt.%)	Si (wt.%)	Mg (wt.%)
1	8.59	28.14	0.91
2	8.06	27.99	0.90
3	7.54	27.03	0.88

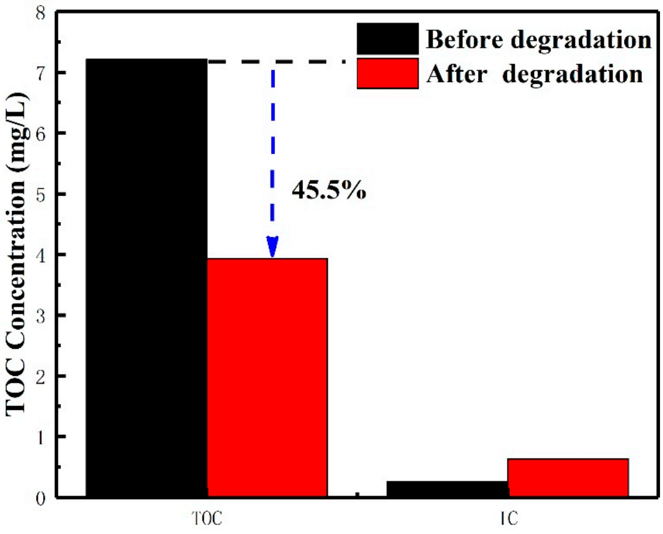

Figure S1. TOC in the B-Mon/PMS system.

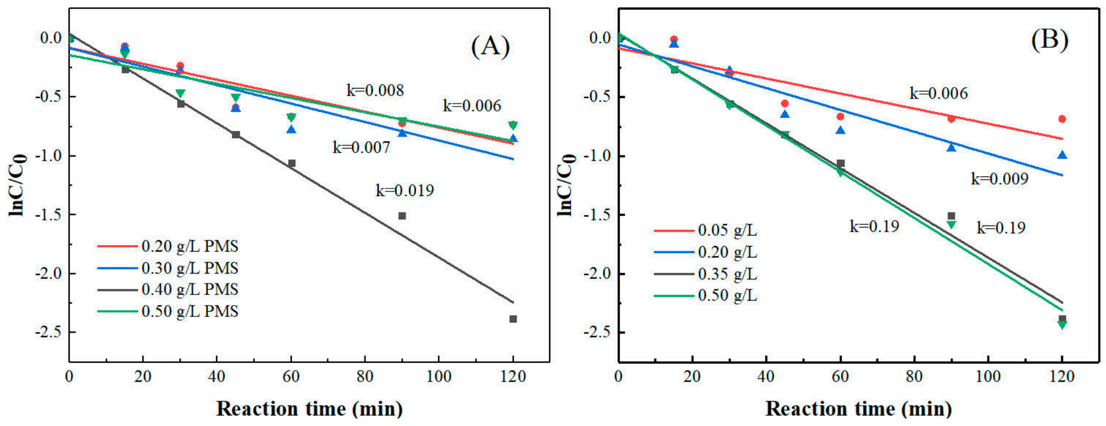

Figure S2. Corresponding reaction rate curves of (A) PMS dosage and (B) catalyst dosage in the B-Mon/PMS system.

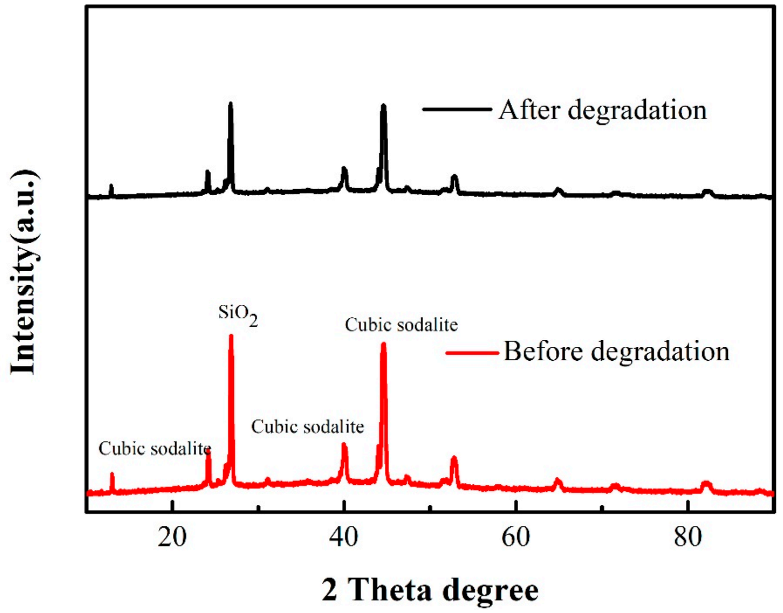

Figure S3. XRD patterns of before and after degradation.

Reference

1. Yang, X.; Yao, X.; Qiu, Y. Introducing and Boosting Oxygen Vacancies within $CoMn_2O_4$ by Loading on Planar Clay Minerals for Efficient Peroxymonosulfate Activation. *Molecules*. **2024**, 29, 3825.
2. Hrichi, A.; Nesrine, A.; Hédi, B.; Marta, P.; Maria, A. Sustainable synthesis of Mangenese cobalt oxide nanocomposite on natural clay and optimization using response surface methodology for ciprofloxacin degradation via peroxymonosulfate activation. *Appl. Sur. Sci.* **2025**, 164136.
3. Jiang, Y.; Cao, X.; Li, F.; Song, X. Influence of Seawater Characteristics on Antibiotic Pollutant Removal via Fe(II)-Peroxymonosulfate-Modified Clay. *Water* **2023**, 15, 1680.
4. Tian, C.; Li, J.; Li, Q.; Nie, Y.; Tian, X.; Dai, C.; Yang, C.; Zhou, Z.; Wang, Y. Surface weak acid-base pair of FeOOH/Al_2O_3 for enhanced peroxymonosulfate activation in degradation of humic substances from water, *Chem. Eng. J.* **2020**, 387, 124064.
